# The shape of human gene family phylogenies

**DOI:** 10.1186/1471-2148-6-66

**Published:** 2006-08-29

**Authors:** James A Cotton, Roderic DM Page

**Affiliations:** 1 Division of Environmental and Evolutionary Biology, Institute of Biomedical and Life Sciences, University of Glasgow, Glasgow, UK; 2 Bioinformatics Laboratory, Department of Biology, National University of Ireland, Maynooth, County Kildare, Ireland

## Abstract

**Background:**

The shape of phylogenetic trees has been used to make inferences about the evolutionary process by comparing the shapes of actual phylogenies with those expected under simple models of the speciation process. Previous studies have focused on speciation events, but gene duplication is another lineage splitting event, analogous to speciation, and gene loss or deletion is analogous to extinction. Measures of the shape of gene family phylogenies can thus be used to investigate the processes of gene duplication and loss. We make the first systematic attempt to use tree shape to study gene duplication using human gene phylogenies.

**Results:**

We find that gene duplication has produced gene family trees significantly less balanced than expected from a simple model of the process, and less balanced than species phylogenies: the opposite to what might be expected under the 2R hypothesis.

**Conclusion:**

While other explanations are plausible, we suggest that the greater imbalance of gene family trees than species trees is due to the prevalence of tandem duplications over regional duplications during the evolution of the human genome.

## Background

**Figure 1:**
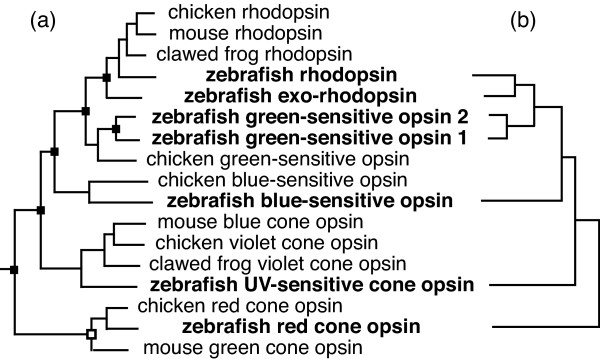
**Gene trees and gene duplications**. Gene family tree for opsins from four vertebrate species – mouse, chicken, zebrafish and clawed frog. (a) including all 4 taxa. Some nodes represent speciation events, others (marked with a black rectangle) gene duplication events. (b) Including only zebrafish sequences. All the nodes in this tree represent gene duplications, so this sort of tree can be used to study the gene duplication process alone

**Figure 2:**
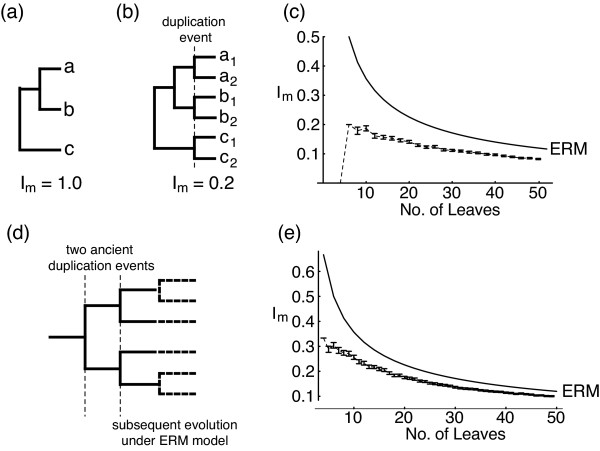
**Effect of large-scale gene duplications on imbalance**. A gene family phylogeny (a) before and (b) after a genome duplication event. I_m _for tree (b) is 0.2. Tree (a) has I_m _1, but expected mean I_m _is 0.5 for evolving to 6 taxa under the ERM model (there is a one-in-three chance of producing a tree as balanced as b). (c) and (e) Show results of two different simulations of genome duplications on trees evolving under the ERM model, showing mean I_m _and 2 standard errors around the mean for 500 trees each from 4 to 50 leaves. (c) Shows the effect of a single, recent genome duplication and (e) the effect of two consecutive ancient episodes of genome duplication (as shown in d). Recent duplications leave a larger signal in I_m _values, despite I_m _giving higher weight to basal branches [43].

Most phylogenetic trees represent the evolutionary history of groups of organisms, with the leaves representing species (or higher taxa) and internal nodes representing speciation events. In contrast, molecular phylogenies for gene families (e.g. figure 1a) usually display sequences for different orthologous groups of proteins [1] from one or more species. These trees can thus show a complicated tapestry of orthology and paralogy, and nodes on such trees may represent either gene duplications or speciations (figure 1, [2]): both are splitting events, producing daughter lineages that henceforth have independent evolutionary histories (at least in the absence of gene conversion or introgression [3]). This similarity between gene duplication and speciation allows similar tools to be used to study the two analogous processes, and techniques developed to investigate speciation and extinction may give some insight into the pattern of gene duplication and gene loss [4,5].

Tree shape has been used to make inferences about the processes of speciation and extinction that govern the birth and death of organismal lineages [6]. We can similarly investigate the processes of gene duplication and gene loss, or deletion, on phylogenies where all the nodes represent gene duplication events, such as those containing homologous genes from a single genome (figure 1). In particular, gene sequences from a completely sequenced genome allow inferences about the process of deletion to be made without confounding this with the absence of a gene from the sequence databases.

### (a) Tree balance, bias and macroevolution

Much of the large literature on tree shape has focused on the balance of trees – how 'comb-like' or 'bush-like' the tree is, ignoring branch length information. In particular, a great deal of work has investigated how the balance of real phylogenies matches that expected under more-or-less simple models of the speciation process [6]. The simplest realistic model is the Equal-Rate Markov model (ERM) or Yule model [7]. Under the ERM model, every lineage has an identical and constant rate of splitting to form new lineages (the actual rate of splitting has no effect). This is often contrasted with the proportional-to-distinguishable arrangements (PDA) model [8], under which every different labelled tree is equally probable. The PDA model may not be realistic [6,9], but is useful because it represents the case in which a tree-building method is selecting randomly from all possible trees. Many models are relaxations of the assumptions of the ERM model, while inaccurate estimation will bias tree shape towards the PDA model.

Previous investigations of tree shape have established that empirical phylogenetic trees are significantly more unbalanced than expected under the ERM model [6]. A number of different explanations for this have been put forward, falling into two categories. The first set of explanations claim that this deviation from the null model is an artefact due either to errors in phylogenetic reconstruction or bias in data collection. Previous work has found that poorly supported maximum-parsimony trees tend to be less balanced than well-supported ones [10], while UPGMA (and presumably other distance-based) trees change little in balance despite being as prone to error as parsimony trees [11], while It seems likely that there is no significant difference between the two for fairly robust data [12]. Mooers [10] demonstrated that complete trees (that include all extant members of a taxon) are more balanced than incomplete trees, as expected if taxon selection across a set of trees is clumped [13]. A second category of explanations claim that deviation from the ERM model accurately reflects that the speciation process is more complex than this model allows. More complex (and perhaps more realistic) models of the speciation and extinction process have been proposed by a number of authors, including Heard [14] and Kirkpatrick & Slatkin [15], who both propose models in which diversification rates evolve through time, producing unbalanced trees, although extremely high rate variation is required to produce the degree of imbalance observed in real data.

### (b) Duplication mechanisms and tree balance

While accurate modelling of species trees has proved complex, the gene duplication process is likely to be even more complicated. Gene duplications within a single gene family are not always independent events, as duplications can occur by a number of different molecular mechanisms [[16] pp. 89–109], some of which copy large quantities of DNA in a single event – duplication by polysomy (the multiplication of a single chromosome pair) and polyploidy (the multiplication of the entire genome) will copy many or all genes in a genome. There is substantial evidence [17,18] that two rounds of whole-genome duplication occurred early in vertebrate evolution (the "2R hypothesis" [16,19]). Duplication of multiple members of a gene family by a single event of these kinds will produce more symmetrical trees than expected under the ERM model if other duplication has occurred at a constant rate (figure 2), or at least shift trees towards greater balance than the underlying process. An equivalent suggestion has been made that balanced species trees may be produced by "synchronous speciation caused by vicariance events that affect most or all of the species in a clade" [15], but whereas large-scale gene duplication is known to occur in a range of different groups, sufficiently large biogeographic events are likely to be rather rare.

**Figure 3:**
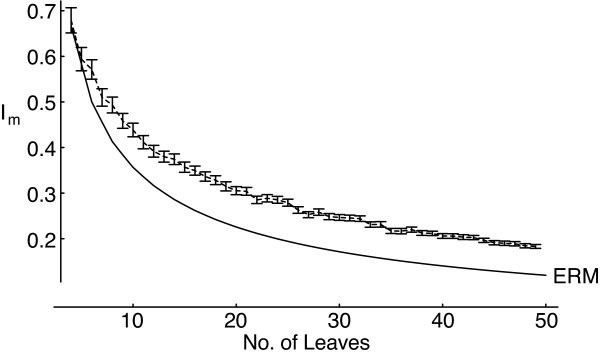
**Effect of tandem duplication on imbalance**. If arrays of tandem duplications duplicate at increasing rates, this could produce highly unbalanced trees. Results of a simulation of a branching process where the probability of a particular branch splitting is proportional to the number of splitting events leading to that branch, based on 500 trees each of sizes from 4 to 50 leaves, showing mean I_m _and 2 standard errors around the mean.

Another peculiarity of gene duplication will have the opposite effect on tree balance, tending to produce less balanced trees. Tandem gene duplications, where a piece of DNA is duplicated adjacent to the original copy, will produce arrays of related genes, such as observed in the developmental Hox gene clusters of metazoans [20]. These repeats of similar sequence will themselves tend to increase the rate at which illegitimate meiotic recombination occurs, and so lead to further tandem duplications [[16] pp. 62–64]. While we know of no suitable quantitative evidence from gene family arrays, this process certainly occurs in minisatellites [21]. Any tendency for the rate of duplication to increase following a duplication will produce unbalanced tree topologies (see figure 3) – it is the opposite situation to that modelled by Losos & Adler [22]. In fact, the problem is rather more complex than this, as only a small proportion of possible tree shapes could actually represent the history of tandem-duplicated genes. While techniques for randomly generating these trees are available [23], it is not clear what the equivalents to the PDA and ERM distributions are for tandem duplication trees.

**Figure 4:**
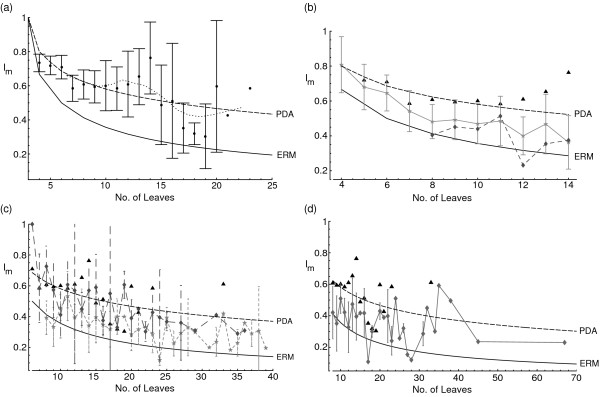
**Imbalance of human gene family trees**. Imbalance of human gene family trees against number of leaves, comparing values for the four different sets of gene family trees used here. (a) Mean I_m _values of human gene families for each leaf number, with bars representing 2 standard errors. Lines connect 10-term moving averages of I_m _values. (b-d) Comparison of imbalance, measured by Colless's I_m_, between human gene family phylogenies (shown as black triangular points) with species phylogenies. (b) Species phylogenies from Heard [31, light gray, unbroken line, star-shaped points] and Mooers [10, mid gray, dashed line, diamond-shaped points]. (c) Morphological phylogenies from Harcourt-Brown [30, mid gray, dashed line, diamond-shaped points] and molecular phylogenies from Harcourt-Brown [29, light gray, dotted line, star-shaped points]. (d) Species phylogenies from Stam [28, unbroken gray line, diamond-shaped points]. Smooth lines on all figures connect expected mean I_m _values under the ERM (lower, solid line) and PDA models (upper, dashed line).

### (c) Shape of gene family phylogenies

In this paper, we make an initial attempt to use tree balance to make inferences about the process of gene duplication. We compare the imbalance of trees for human gene families with expectations from the ERM model and with the imbalance of species phylogenies collated by other workers. This latter comparison is useful because of the uncertainty surrounding how tree construction might affect balance: to the extent that both are constructed from similar data, differences between the balance of gene trees and species trees will be due to differences in the branching processes of duplication and speciation. We can effectively use the species tree data as an informal control to highlight whether the balance of gene trees requires explanation in terms of the mechanism of gene duplication. Previous work on the imbalance of gene family trees looked exclusively at using four-member gene families to test the 2R hypothesis. In the absence of other duplication and gene deletion, two consecutive genome duplications should amplify a single gene into a 4-member gene family with a perfectly balanced tree topology [24,25]. Hughes [25] and Martin [24] both found that most 4-member gene families are unbalanced, and hence rejected the 2R hypothesis. Our dataset enables us to put these earlier results in context by comparison with gene families of other sizes.

## Results

The single-linkage clustering approach divided the protein-coding genes from the human genome into 17,908 gene families (including families of a single gene, see methods). The distribution of gene family sizes was consistent with previous work [26]. Trees were constructed for 1,265 gene families with reasonable alignments for more than 3 members. Colless's index is undefined for polytomous trees, and 550 trees were excluded because they contained at least one zero-length internal branch, leaving a total dataset of 715 gene families.

Figure 4 shows the imbalance of our trees in comparison with expected values under the ERM and PDA models. Clearly, gene family trees are more unbalanced than expected under the ERM model but substantially more balanced than expected under the PDA model. This can be confirmed for the ERM model because the individual _p_I_m _scores can be combined using Fisher's method to yield an overall p-value that the trees have been drawn from an ERM distribution [[27] pp.794–797], which is significantly rejected for our data (χ^2 ^= 1693.9, df = 1430, P < 0.0001). A test of shape for unrooted trees [28] confirms that the gene family trees are significantly less balanced than expected under the ERM and do not fit the PDA distribution, showing that these results are not simply due to the rooting information (for ERM, 1-tailed test, χ^2 ^= 2456.4, df = 1430, P < 0.0001; for PDA, 2-tailed test, χ^2 ^= 2120.6, df = 1430, P < 0.0001).

Gene family data were compared with existing datasets of species phylogenies using GLMs as described in Methods. These data support the finding of Stam [29] that transformed _p_I_m _scores are not independent of tree size. Using the categorical models as described, our data were found to be significantly more unbalanced than real trees for two out of three incomplete-tree datasets for which full information was available [30] (for Harcourt-Brown 2002 data [100 molecular trees], GLM of arcsine-transformed _p_I_m _scores with number of leaves and dataset as factors: Nleaves (number of taxa) F = 12.038, df = 34/814, P < 0.0001, dataset F = 8.35, df = 1/814, P = 0.0039; Harcourt-Brown 2001 data [100 morphological trees], GLM of arcsine-transformed _p_I_m _scores with number of leaves and dataset as factors: Nleaves F = 14.962, df = 27/814, P < 0.0001, dataset F = 0.7939, df = 1/814, P = 0.373; for Heard 1992 data [249 trees], GLM of arcsine-transformed _p_I_m _scores with number of leaves and dataset as factors: Nleaves F = 22.992, df = 19/963, P < 0.0001, dataset F = 6.470, df = 1/963, P = 0.0111). Imbalance measures for all of these sets of trees are shown on figure 4. The only dataset that is not significantly different from our gene family trees by this test is that of morphological trees from Harcourt-Brown [31]. This is probably because half of the trees from this dataset include fossil taxa, which make these trees more unbalanced than trees containing only contemporaneous leaves [31].

Data for complete trees was available from two different compilations [10,29]. Comparison with Mooers [10] data is difficult as neither topologies or I_m _scores for individual trees were available, but our gene family trees are more unbalanced than the ones compiled by Mooers (median _p_I_m _scores for his data, with Nleaves from 8–14, is 0.429, gene family trees of 8–14 leaves,152 trees with median _p_I_m _score 0.246). Stam [29] collected a larger set of 69 complete species trees, including larger trees than Mooers. A statistical test as above confirms that human gene family trees show significantly less balance than the trees collected by Stam (GLM of arcsine-transformed _p_I_m _scores with number of leaves and dataset as factors: Nleaves F = 13.097, df = 32/783, P < 0.0001, dataset F = 10.866, df = 1/783, P = 0.0010).

For all the GLMs summarised above, there was no significant interaction between tree size and dataset, so the interaction term was not included in any analysis. Treating tree size as continuous, an 8th-order polynomial was needed to model the relationship between tree size and _p_I_m _(terms up to the 8th power of Nleaves were significant, terms with higher powers were not). These analyses gave identical results to those reported above (treating tree size, Nleaves, as categorical) in that it made no difference to the significance or otherwise of any terms in the models, and confirms the direction of the effect – that gene family trees are less balanced than species trees.

For four-member gene families, 215 out of 293 gene families, or 73%, are unbalanced, while two-thirds of such trees should be unbalanced under the ERM model. Tests based on _p_I_m _scores have little power for small gene families, but a binomial test confirms that significantly more trees are unbalanced than the ERM expectation (n = 293, prob. = 2/3, P = 0.0055), reflecting the general trend of human gene family trees.

## Discussion

Human gene family phylogenies are more unbalanced than species trees compiled from the literature and than expected under the ERM model, suggesting that the process of gene duplication occurs similarly, but not identically, to that of speciation. This difference in balance may be due to different biases acting on gene trees than on species trees. Taxon sampling seems unlikely to be the explanation as our gene family trees are complete (they sample all extant genes in the human genome) and our gene family trees are more unbalanced than complete species trees. The difference between our trees and published cladograms is unlikely to be due to differences between morphological and molecular data as our trees are less balanced than a compilation of molecular trees [30] and studies have found no significant difference between the balance of trees from morphological and molecular data [30,32]. It seems that the imbalance of gene family trees demands a mechanistic rather than methodological explanation.

Many differences between the processes of gene duplication and speciation might explain the different shapes of the trees produced and, in principle, any of the models that have been invoked to explain deviations of observed species trees from ERM expectations could be acting on gene duplications to a greater extent. For example, if the model of evolving rates suggested by Heard [14] applied to duplication rates with greater variation than for speciation rates, this would predict the sort of difference observed. It is unclear what molecular mechanism could cause this. Given that the processes of regional, large-scale gene duplication and tandem duplication through recombination are known to occur in genomes, we interpret our results in terms of these mechanistic models.

Seen in this light, the high imbalance of gene family trees suggests that large-scale duplication has not played a sufficiently large role in gene family evolution to leave any signal in the cladistic balance of gene family trees, or that the rate of gene shuffling after tandem duplication is high enough to move duplicated genes apart before regional duplication occurs [33]. The continuous process of duplication and loss that appears to have occurred during the evolution of many genomes [4,34] produces highly imbalanced gene family trees. Our data do not provide a powerful test of the 2R hypothesis, as gene deletion may have erased any trace of this event from many of our gene families, particularly if massive gene loss quickly followed the polyploidy events [35]. Similarly, it is not surprising that the balance of four-taxon trees from our data supports previous work [24,25] as we would expect these trees to be shaped by a variety of gene duplication events and show similar imbalance to larger gene families.

If many of our gene families do sample duplications from the 2R event, our results are even more striking, as gene family trees are highly imbalanced despite this large event, but there are further caveats. The simulations of figure 2 are probably an inadequate model of how the 2R event occurred: Furlong and Holland [36] have suggested that the two genome duplications of the 2R-event may have been closely spaced in time, leading to a period of auto-octoploidy. This octoploid genetic system would break down through a gradual and random return to diploidy as chromosomes pair increasingly preferentially with particular other homologues. In this case, the phylogeny of the duplicated genes will reflect the process of diploidisation rather than the pattern of polyploidisation that produced the duplicate copies. For example, if diploidisation occurred by pairs of homeologues diverging from the pool of chromosomes sequentially, it could lead to imbalanced gene family trees, while if it occurs through a pseudo-tetraploid intermediate stage, it would tend to produce balanced topologies. If Furlong and Holland are right, and the two '2R' events occurred almost consecutively, then the balance of gene families will be a product of the background duplication process superimposed over the signal from the diploidisation process, which will be hard to disentangle.

We have identified two processes – large-scale gene duplication and tandem duplication – that have shaped gene family phylogenies and do not apply in the analogous process of speciation. More sophisticated models of regional gene duplication would show the different effects that the size, number, and timing of such events could have on the balance of phylogenetic trees, as we have modelled only two very simple situations (figures 2 and 3). The balance of gene family trees may reflect the relative rates of large-scale duplication and tandem duplication, but other processes can also affect tree balance. While tree balance provides a method to study these processes, further progress will also require a better understanding of the background birth-death processes of speciation and extinction and gene duplication and loss.

## Conclusion

Gene family trees are significantly less balanced than would be expected under the equal-rate Markov (ERM) model and are even more unbalanced than published species trees. The different balance of gene family trees and species phylogenies suggests some difference between the processes of gene duplications and speciation. This difference is surprising, as regional duplication is known to occur, leading to non-independent gene duplications, which should produce more balanced trees. The imbalance of gene family trees suggests that relatively few gene duplications have occurred as segmental duplications affecting multiple loci. Some models of tandem duplication suggest that this process should produce unbalanced gene family trees, so our results might indicate that tandem duplications are more common, or at least have had a greater impact on the shape of gene trees. One important complication is the uncertainty over the effect the 2R event could have had on the evolution of vertebrate gene families.

## Methods

Additional material is available from [37]. Available from this site is a text file listing the number of taxa and Colless's index of imbalance for each family, a Mathematica notebook for calculating expected values of this index under the ERM and PDA models and tables of expected values for Colless's Index under these models for trees of between 3 and 500 leaves. C++ code for generating the distribution of Colless's Index under ERM and PDA models and for the simulations shown in the paper is also available, as are full results for the statistical tests described.

### a) Building gene family trees

The *blastclust *program [38] was used to form single-linkage clusters for all human genome genes using amino-acid sequences from the NCBI reference sequence of 15/05/2006, with sequences linked if they have a mean bit score of 0.75 over at least 50% of each sequence (-S 0.75 -L 0.5 -b T options of *blastclust*). These sequences were then matched with invertebrate outgroups by blast searches against the entire invertebrate section of Genbank. A database of all the sequences was compared with the outgroup database using the *blastp *option of the *blastall *program, taking the two best hits per sequence with expectation (E-value) less than 0.1. Alignments were generated for all families with more than 3 and less than 500 member sequences using ClustalW [39] with default parameters. Short sequences (less than 30% the mean length for a family, and families with aligned length less than 50 residues) were removed, and phylogenetic trees constructed using the neighbour-joining algorithm [40] on maximum-likelihood distances inferred using Tree-Puzzle v5.0 [41] under the model selected by the program. Gene families were taken as subtrees containing only human sequences, and so contain paralogs generated since the last common ancestor of the vertebrates. Midpoint rooting of our trees produced phylogenies more balanced than outgroup rooting, suggesting that the outgroups are not too distant from the ingroup taxa for accurate rooting. Some families were discarded due to difficulties in alignment or tree reconstruction. Trees were rooted using the outgroups and Colless's I_m _was calculated using a C++ program.

### b) Simulating genome duplications

To establish the effect that non-independent gene duplication has on tree balance, the effect of the most extreme non-independent event, a whole-genome duplication, was simulated. A C++ program was used to evolve trees under the ERM model but with every lineage duplicating simultaneously as the final cladogenesis event (Figure 2a, b). A separate simulation simulated two consecutive genome duplications as the first cladogenesis events in a gene family, followed by subsequent evolution under the ERM model (Figure 2d).

### c) Colless's index

The most widely used index of tree imbalance is Colless's [42] coefficient of imbalance (I_m_) and it has proved to be mathematically tractable [43].This index takes the sum, over every node in the tree, of the absolute difference in the number of leaves descended from its two descendant nodes. I_m _is usually normalised to range from 0 (for a completely balanced topology) to 1 (for a completely unbalanced topology) by dividing by (n−1)(n−2)2
 MathType@MTEF@5@5@+=feaafiart1ev1aaatCvAUfeBSjuyZL2yd9gzLbvyNv2CaerbuLwBLnhiov2DGi1BTfMBaeXatLxBI9gBaerbd9wDYLwzYbItLDharqqtubsr4rNCHbGeaGqipu0Je9sqqrpepC0xbbL8F4rqqrFfpeea0xe9Lq=Jc9vqaqpepm0xbba9pwe9Q8fs0=yqaqpepae9pg0FirpepeKkFr0xfr=xfr=xb9adbaqaaeGaciGaaiaabeqaamaabaabaaGcbaWaaSaaaeaacaGGOaGaamOBaiabgkHiTiaaigdacaGGPaGaaiikaiaad6gacqGHsislcaaIYaGaaiykaaqaaiaaikdaaaaaaa@3E82@, where *n *is the number of leaves on the tree. Recursion equations for the probability distribution of this measure under both the ERM and PDA models are available [43][44]. I_m _is a powerful statistic for trees from a variety of models but different measures have different properties [6][45][46], and may be more useful in different situations. Despite this, to allow easy comparison with previous data sets we only employ the normalised form of Colless's I_m _in this paper.

### d) Statistical tests of imbalance

In common with previous workers, statistical tests were based on _p_I_m _scores [15,32]. To calculate these scores, each tree's I_m _was compared with that of 10,000 trees of the same size simulated under the ERM model, and the _p_I_m _score was taken as the fraction of these with the same or more extreme I_m _scores: i.e. the p-value of observing this unbalance under the ERM model. To ensure greater homogeneity of variance within tree sizes, _p_I_m _scores used in statistical tests were transformed using the arcsine transformation [[27] p.421]. Statistical tests used here include the number of leaves in the model as _p_I_m _scores are not independent of tree size [29]. As the relationship between _p_I_m _and number of leaves is non-linear we adopt a conservative method, treating number of leaves as categorical so that _p_I_m _values are only compared for trees of the same size. An alternative approach, treating number of leaves as continuous, and using a suitable polynomial to fit the non-linear relationship between _p_I_m _and number of leaves was also used to ensure that statistical significance was not an artefact of the statistical model. An equivalent and more elegant approach would be use the I_m _statistic re-normalised to be independent of tree size for data from the Yule distribution [47].

## Authors' contributions

JAC designed and carried out the study, performed statistical analysis and drafted the manuscript. RDMP helped design the study and draft the manuscript. Both authors read and approved the final manuscript.
